# Biomechanical comparison of all‐inside meniscal suture configurations for posterior root tear: Three conventional stitches versus delta‐grip stitch

**DOI:** 10.1002/jeo2.70149

**Published:** 2025-01-22

**Authors:** Kyohei Ishibashi, Kyota Ishibashi, Takahiro Tsushima, Eiji Sasaki, Shohei Yamauchi, Yuka Kimura, Yasuyuki Ishibashi

**Affiliations:** ^1^ Department of Orthopaedic Surgery Hirosaki University Graduate School of Medicine Hirosaki Japan

**Keywords:** all‐inside repair, cinch stitch, delta‐grip stitch, medial meniscus posterior root tear

## Abstract

**Purpose:**

This study aimed to compare the biomechanical properties of four meniscal suture configurations—two simple sutures (TSS), two cinch sutures, a locking loop stitch (LLS), and a delta‐grip stitch (DGS)—for transtibial pullout repair of medial meniscus posterior root tears (MMPRTs) using porcine menisci.

**Methods:**

Forty porcine menisci were randomly assigned to each suture configuration with all‐inside repair. All specimens were subjected to cyclic loading for 1000 cycles, followed by a load‐to‐failure test. We evaluated displacement after cyclic loading, the ultimate failure load and the mode of failure.

**Results:**

No significant differences in displacement were observed between the configurations (*p* = 0.709). The DGS exhibited significantly higher ultimate failure loads (281.4 ± 53.5 N) compared to TSS (166.8 ± 84.5 N) and the LLS (119.7 ± 46.7 N) (*p* = 0.006 and *p* < 0.001, respectively). Suture breakage was observed in the DGS group, while meniscus cutout was observed in the other suture configuration models.

**Conclusion:**

The results suggest that the DGS provides superior fixation strength and enhances MMPRT repair outcomes.

**Level of Evidence:**

Level IV.

**Abbreviations**
DGS: delta‐grip stitch
LLS: locking loop suture
MMPR: medial meniscal posterior root
MMPRT: medial meniscal posterior root tear
TCS: two cinch sutures
TSS: two simple sutures

## BACKGROUND

The meniscus is a crescent‐shaped fibrocartilaginous structure that plays a crucial role in stabilizing complex knee kinetics such as tension, compression and shear stress [6, 19]. This structure distributes contact stress to the tibial plateau as circumferential hoop stress.

Medial meniscal posterior root (MMPR) tears (MMPRTs) have been increasingly recognized as impairing the transmission of circumferential hoop stress [1, 10, 15]. In MMPRTs, the critical biomechanical functions of the meniscus are disrupted, leading to accelerated arthritic degeneration, abnormal loading, instability and the potential need for knee arthroplasty, particularly in younger patients [12, 17, 21]. If an MMPRT remains untreated, it is likely to lead to results similar to those of total meniscectomy, potentially necessitating total knee arthroplasty [13, 14, 16]. These findings suggest the necessity of MMPRT repair to restore normal knee function. Currently, transtibial pullout repair and suture anchor methods are recommended to repair MMPRTs [3, 7, 11, 12].

Several arthroscopic suture techniques have been reported for transtibial pullout repair, including two simple sutures (TSS), two cinch sutures (TCS) and locking loop suture (LLS) mattress sutures [7, 8, 17]. These procedures are technically demanding and are performed in a narrow medial joint space. Strong MMPR fixation is crucial to avoid early failure. To address these challenges, we developed a new technique, namely, the delta‐grip stitch (DGS), using an all‐inside passer [9]. This technique provides easy access to the anterior portal and a strong grasp of the MMPR. However, it remains unclear whether the tensile strength of the DGS is higher than that of conventional configurations.

This study aimed to compare the failure load of the DGS with that of conventional suture techniques for transtibial pullout repair in MMPRTs. We hypothesized that the DGS would provide a higher failure load than the other configurations.

## METHODS

### Specimen preparation

Forty medial menisci from pigs were used in this study. The menisci were sharply resected from the knee joint, and the attachment fibres were carefully cut from the menisci. The medial menisci were frozen at −20°C immediately after harvesting and thawed overnight at 4°C prior to testing. The menisci were randomly assigned to one of the four suture techniques used for transtibial pullout repair of the MMPRTs.

### Suturing configurations

**Figure 1:**
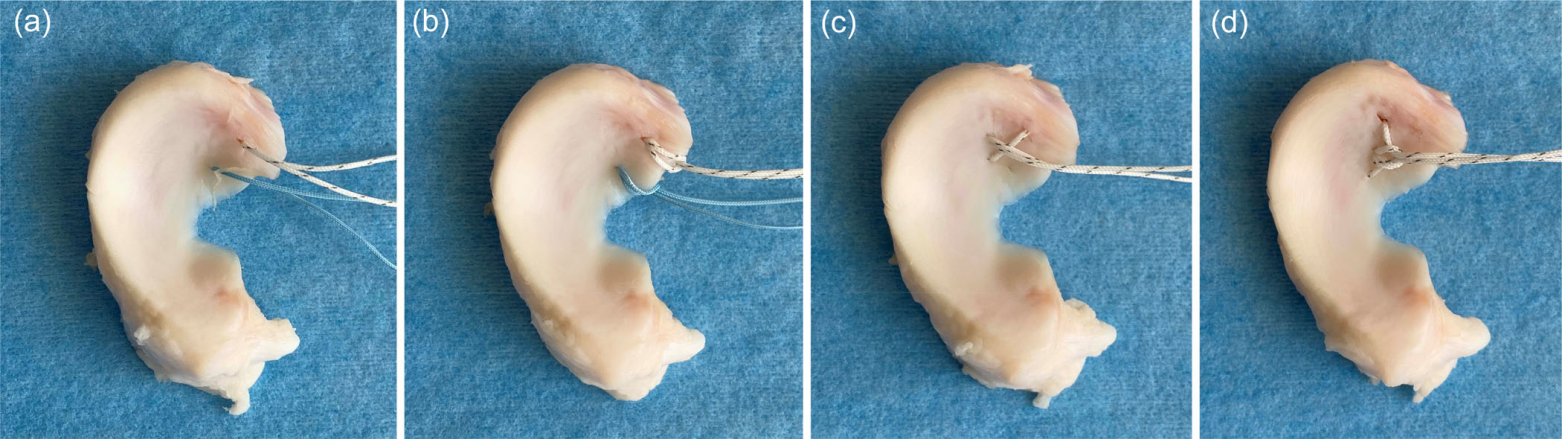
Suture configurations. (a) two simple stitches, (b) two cinch stitches, (c) locking loop stitch and (d) delta‐grip stitch.

We compared four meniscal suture configurations: TSS, TCS, LLS and DGS (Figure 1). Ten menisci were assigned to each group. A trained orthopaedic surgeon performed all of the meniscal suturing. No. 2 FiberWire sutures (Arthrex) were used for all suture configurations along with a self‐retrieving suture passer device (FIRSTPASS MINI Suture, Smith & Nephew).

The TSS technique was performed by passing two sutures 5 mm medial to the medial MMPR (Figure 1a). For the TCS, the middle of the suture was placed in the suture passer and passed 5 mm medial to the MMPR. The two free ends of the suture were passed through the loop [14] (Figure 1b). The LLS technique was performed by passing a single horizontal mattress suture through the meniscus, with the free suture ends passing over the loop (Figure 1c). The DGS technique was performed as follows [9]: one end of the suture was passed through the posterior corner of the MMPR from the distal to the proximal side. The middle of the suture was passed through the middle of the MMPR in a loop, from the inferior to the superior side. The remaining end of the suture was drawn through the opposite corner of the MMPR, from the inferior to the superior side. Both ends of the suture were passed through the loop at the centre of the suture and the loop was tightened, similar to a cinch stitch (Figure 1d).

### Biomechanical testing

**Figure 2:**
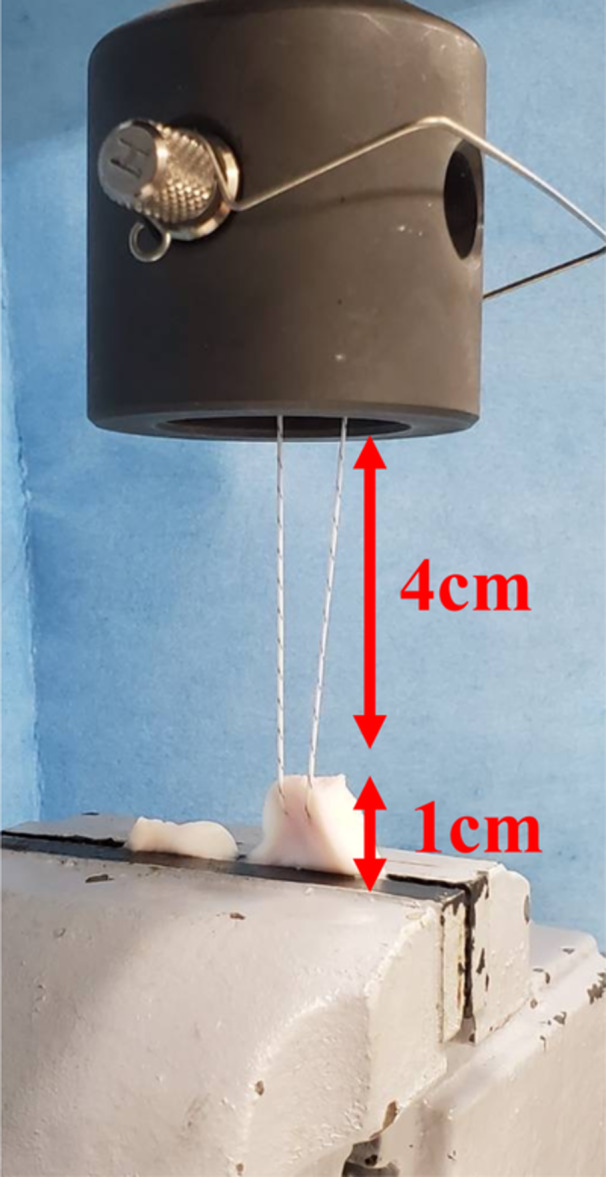
Set‐up for biomechanical testing. The sutures were fastened to the upper clamp. Each meniscus was attached to the lower clamp at a point 1 cm medial to the medial meniscus posterior root.

Biomechanical testing was performed using a material‐testing machine (Instron 4465; Instron). Each meniscus was attached to the lower clamp 1 cm medial to the medial MMPR. The sutures were fastened at the distal end, 4 cm from the MMPR, to the upper clamp (Figure 2). A cyclic loading test was performed with a loading force between 5 and 20 N at a rate of 200 mm/min for 1000 cycles. The displacement caused by the cyclic test was defined as the difference between the distances measured in the first and last cycles. After the cyclic loading tests, load‐to‐failure tests were conducted at a displacement rate of 30.0 mm/s. The ultimate failure loads and modes were recorded.

### Statistical analysis

A post hoc power analysis was performed to determine the power of the present study. Based on the means and standard deviations of the failure loads, an effect size of 0.76 was calculated. With this effect size, a power of 0.80 was achieved with eight samples per group.

A one‐way analysis of variance was performed to compare the overall effect of the suture technique on the displacement and ultimate failure load, followed by a Tukey's post hoc test to detect significant differences. Statistical analyses were performed using SPSS software for Windows (version 21.0; SPSS). The statistical significance threshold was set at a *p* value of 0.05.

## RESULTS

**Figure 3:**
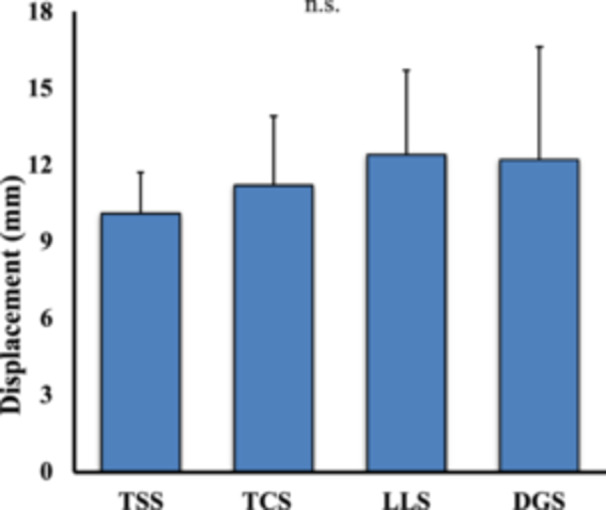
Displacement after cyclic load. Comparison of the displacement distances for each suture configuration DGS, delta‐grip strength; LLS, locking loop stitches; TCS, two cinch stitches; TSS, two simple stitches.

No suture breakage was observed during the cyclic loading tests. The mean displacement was 10.1 ± 1.6, 11.2 ± 3.3, 12.4 ± 3.3 and 12.2 ± 4.4 mm in the TSS, TCS, LLS and DGS groups, respectively (Table 1). There were no significant differences in the displacement distances between the four suture configurations after cyclic loading (*p* = 0.709) (Figure 3).

**Table 1:** Ultimate failure load of each suture configuration (N).

	Mean (N)	Deviation (N)	95% CI	Minimum	Maximum
TSS	166.8	84.5	88.7–244.9	93.4	336
TCS	119.7	46.7	76.6–162.9	68.8	181.5
LLS	213.2	92.4	142.2–284.2	103.7	330.1
DGS	281.4	53.5	250.5–312.3	199.6	345.8

*Note*: These values represent the ultimate failure loads for each suture configuration.

Abbreviations: 95% CI, 95% confidence interval; DGS, delta‐grip strength; LLS, locking loop stitches; TCS, two cinch stitches; TSS, two simple stitches.

**Figure 4:**
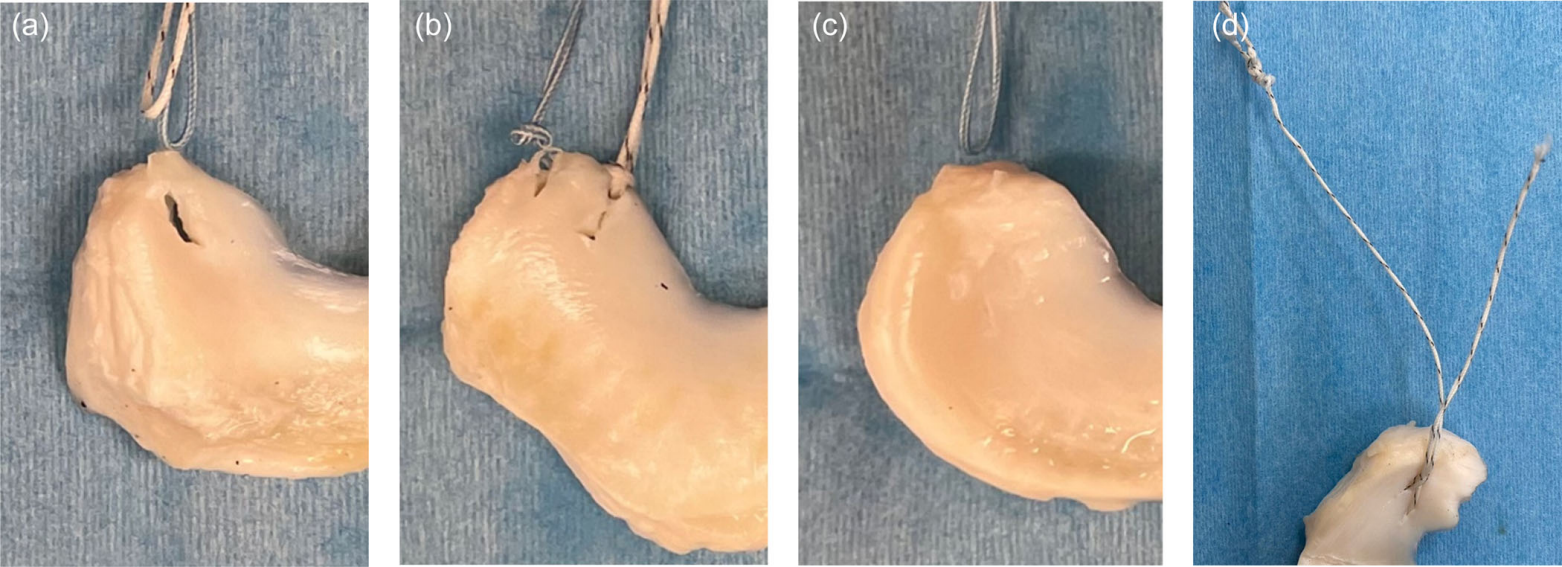
Mode of failure. (a) The mode of failure for two simple stitches, (b) two cinch stitches, (c) locking loop stitch and (d) delta‐grip stitch.

**Figure 5:**
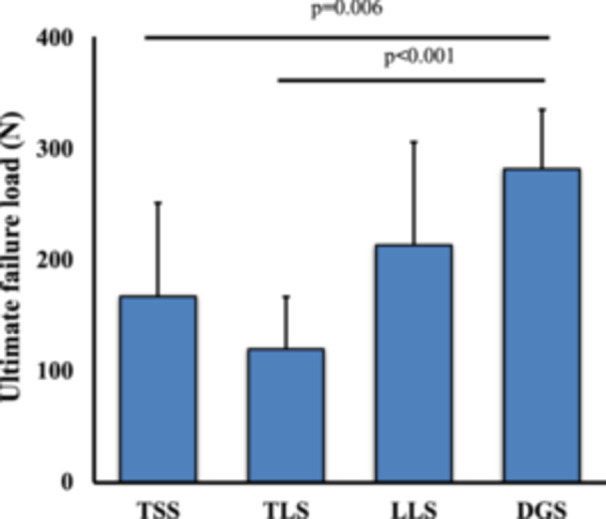
Ultimate failure load. Comparison of the ultimate failure load for each suture configuration. DGS, delta‐grip strength; LLS, locking loop stitches; TCS, two cinch stitches; TSS, two simple stitches.

Suture breakage was observed in the DGS group, while meniscus cutout was observed in the other suture configuration models (Figure 4). The mean ultimate failure load was 166.8 ± 84.5, 119.7 ± 46.7, 213.2 ± 92.4 and 281.4 ± 53.5 N in the TSS, TCS, LLS and DGS groups, respectively. The ultimate failure load was significantly higher in the DGS group than in the TSS and TCS groups (*p* = 0.006 and *p* < 0.001, respectively). No significant difference was observed in the ultimate failure load between the LLS and DGS groups (Figure 5).

## DISCUSSION

This study revealed that the DGS exhibited a significantly higher ultimate failure load than the TSS and TCS configurations. No significant difference in failure load was observed between the DGS and LLS groups; however, meniscus cutout was not observed in the DGS group. These results supported our hypothesis that the new DGS configuration would provide a superior fixation strength. While the load types that apply to the repaired root during knee motion are not unknown, we consider that the increased ultimate failure load of the DGS may improve MMPRT healing.

Determining the biomechanical properties of meniscal repair techniques is crucial in clinical practice because they directly influence the success and durability of the repair. Various suturing techniques have been reported for repairing MMPRTs. Okimura et al. reported that the failure load of MMPRT repair using the FastFix 360 device (Smith & Nephew Endoscopy) did not differ significantly from that using a single simple suture and the TSS technique [20]. Fujii et al. reported that a modified Mason‐Allen suture combined with Ultrabrade (Smith & Nephew Endoscopy) exhibited a higher failure load than the TSS technique in a porcine meniscus study [7]. These studies have prompted the development of simpler and stronger suturing techniques.

The DGS provides a simple locking suture using an all‐inside device [9]. Both the DGS and the Mason‐Allen stitch pass the suture through the meniscus horizontally, while the TSS, TCS and LLS techniques involve passing the suture orthogonally to the meniscus [4, 18]. Previous studies have reported that the Mason‐Allen stitch has better biomechanical properties with regard to cyclic load and failure load than the TSS, TCS and LLS [2, 5]. In this study, the DGS showed better mechanical properties than the TSS and TCS. In our clinical practice, we perform a cinch stitch alongside the DGS to prevent meniscus cutout. We have had no experience with meniscus cutouts occurring immediately after performing the DGS technique (unpublished data). Our study contributes to existing literature by evaluating a novel suturing technique and comparing it with established methods. The higher ultimate failure load observed with the DGS suggests its potential as a reliable option for MMPRT repair, offering enhanced stability and a reduced risk of failure.

After the cyclic loading test, each group showed an elongation of approximately 1 cm. However, not statistically significant, elongation in the LSS and DGS groups was larger than that in the TSS group. This may be because of the tightening of the loop over the meniscus during cyclic loading. A 1 cm elongation would imply disruption of MMPRT repair and the MMPR would be unable to heal to the attachment site. Clinically, it is important to remove any slack from the loop prior to fixing the tibial side. Furthermore, rehabilitation before MMPRT healing should be avoided.

The minimum failure load was considerably greater in the DGS group than in the other groups, being two times as much, and the other groups all had failure loads around 100 N in the worst case. This finding is consistent with high failure rates reported in previous studies [3, 11]. Removing slack prior to the final fixation is essential; however, if the minimum failure load is small, excessive traction force cannot be applied. Therefore, the high minimum failure load is a further major advantage of the DGS technique.

Despite these promising findings, this study had several limitations. Porcine menisci were used, which may not perfectly replicate the biomechanical characteristics of human menisci. While the use of animal models provides valuable insights, extrapolating these results directly to clinical practice requires caution. Future studies incorporating human cadaveric specimens and clinical trials are warranted to validate our findings in human subjects. The sample size was relatively small, with only 10 specimens per group. We conducted sample size calculations to ensure adequate power, but larger sample sizes would enhance the robustness and generalizability of our conclusions. Additionally, variations in tissue quality and anatomical differences among specimens may have influenced the results. Further research with larger sample sizes and standardized experimental conditions is required to validate our findings. This study focused on biomechanical outcomes, overlooking other important factors, such as surgical ease, postoperative rehabilitation and clinical outcomes such as pain relief and functional improvement. Moreover, this study did not account for fixation on the tibial side, which may have also influenced failure outcomes. The potential friction between the knot and the condyle, as well as the risk of chondral damage owing to knot placement, was not evaluated in this analysis. Future studies should consider these factors to provide a more comprehensive assessment of suture performance in clinical scenarios. A comprehensive evaluation of these factors is essential to assess the overall efficacy and feasibility of the DGS technique compared with conventional techniques.

In conclusion, our study highlights the potential of using the DGS technique as a promising option for transtibial pullout repair of MMPRTs, offering superior fixation strength compared with conventional suture configurations. However, further research addressing the limitations of this study is necessary to establish the clinical relevance and applicability of the DGS in the management of MMPRTs.

## AUTHOR CONTRIBUTIONS

Kyohei Ishibashi participated in the study design, supervised data collection and drafted the manuscript. Kyota Ishibashi, Takahiro Tsushima and Shohei Yamauchi participated in data collection. Eiji Sasaki participated in data collection, assisted with statistical analysis and drafting of the manuscript and provided critical revision of the manuscript. Yasuyuki Ishibashi conceived and designed the study, supervised the team, facilitated acquisition of the data and provided direction for and critical review of the manuscript. All authors read and approved the final manuscript.

## CONFLICT OF INTEREST STATEMENT

The authors declare no conflicts of interest.

## ETHICS STATEMENT

All procedures involving human participants performed in this study were in accordance with the ethical standards of the institutional and/or national research committee and with the 1964 Helsinki Declaration and its later amendments or comparable ethical standards. This study was approved by our institution's internal review board. All applicable international, national, and institutional guidelines for the care and use of animals were followed.

## Data Availability

The data sets generated and/or analyzed during the current study are not publicly available but are available from the corresponding author on reasonable request.
